# Factors Affecting Recruitment of Participants for Studies of Diabetes Technology in Newly Diagnosed Youth with Type 1 Diabetes: A Qualitative Focus Group Study with Parents and Children

**DOI:** 10.1089/dia.2016.0157

**Published:** 2016-09-01

**Authors:** Conor Farrington, Janet Allen, Martin Tauschmann, Tabitha Randell, Nicola Trevelyan, Roman Hovorka

**Affiliations:** ^1^ Cambridge Centre for Health Services Research, Institute of Public Health, School of Clinical Medicine, University of Cambridge, Cambridge, United Kingdom.; ^2^ Department of Paediatrics, Wellcome Trust-MRC Institute of Metabolic Science, University of Cambridge, Cambridge, United Kingdom.; ^3^ Department of Paediatric Endocrinology and Diabetes, Nottingham Children's Hospital, Nottingham University Hospitals NHS Trust, Nottingham, United Kingdom.; ^4^ Department of Paediatric Endocrinology and Diabetes, Southampton Children's Hospital, University Hospital Southampton NHS Trust, Southampton, United Kingdom.

## Abstract

***Background:*** Relatively little is known about parents' or children's attitudes toward recruitment for, and participation in, studies of new diabetes technologies immediately after diagnosis. This study investigated factors affecting recruitment of participants for studies in newly diagnosed youth with type 1 diabetes.

***Methods:*** Qualitative focus group study incorporating four recorded focus groups, conducted in four outpatient pediatric diabetes clinics in large regional hospitals in England. Participants comprised four groups of parents (*n* = 22) and youth (*n* = 17) with type 1 diabetes, purposively sampled on the basis of past involvement (either participation or nonparticipation) in an ongoing two-arm randomized trial comparing multiple daily injection with conventional continuous subcutaneous insulin infusion regimens from the onset of type 1 diabetes.

***Results:*** Stress associated with diagnosis presents significant challenges in terms of study recruitment, with parents demonstrating varied levels of willingness to be approached soon after diagnosis. Additional challenges arise regarding the following: randomization when study arms are perceived as sharply differentiated in terms of therapy effectiveness; burdens arising from study participation; and the need to surrender new technologies following the end of the study. However, these challenges were mostly insufficient to rule out study participation. Participants emphasized the benefits and reassurance arising from support provided by staff and fellow study participants.

***Conclusions:*** Recruitment to studies of new diabetes technologies immediately after diagnosis in youth presents significant challenges, but these are not insurmountable. The stress and uncertainty arising from potential participation may be alleviated by personalized discussion with staff and peer support from fellow study participants.

## Introduction

Anumber of challenges surround studies of new diabetes technologies in youth, especially as children's involvement in medical research is likely to include the involvement of, and in many cases obtaining consent from, parents or legal guardians. Children's participation in medical research raises important ethical issues, including children's inherent vulnerability and, for adolescents in particular, the challenges arising from major psychological and physiological changes occurring during research participation.^1^ Previous research has also emphasized the complexity of parents' roles in terms of their children's research participation. A number of tensions arise in this context, such as that between parents seeking to take responsibility for their child's well-being, on one hand, and surrendering their child's well-being to medical staff during trials and/or standard care, on the other hand.^2^ Research has shown that parents who lack understanding of research concepts such as randomization may be less likely to allow their children to participate in randomized trials and that parents of children randomized to control arms of randomized trials experience significant levels of disappointment—one of many factors that can limit future research participation.^2–7^

For parents of children with newly diagnosed type 1 diabetes, these general issues surrounding research participation are supplemented by a number of condition-specific challenges. Randomized trials investigating the impact of technological treatments on disease progression frequently necessitate the recruitment of children immediately after diagnosis. However, this period is a highly stressful time for children and parents, typically occurring with little warning and requiring multiple significant life changes in a very short time frame, with the threat of significant long-term morbidity, including micro- and macrovascular complications.^8,9^ One study indicated that 51% of mothers and 41% of fathers of children newly diagnosed with type 1 diabetes met some or all of the diagnostic criteria for posttraumatic stress disorder as defined by the Diagnostic and Statistical Manual of Mental Disorders (DSM-IV).^10^ Parental stress arises from a number of possible sources, including traumatic events (e.g., hospitalization) surrounding the onset of type 1 diabetes, the threat of significant morbidity arising from the condition, and parental responsibility in terms of caring for a child with diabetes.^10^ The self-care regimens required for intensive insulin therapy are complex and can lead to diabetes-based parent–child conflict, particularly when parental involvement in care (e.g., to reduce the risk of hypoglycemia) conflicts with adolescents' developing sense of autonomy.^11^

Thus, both generic and condition-specific challenges confront parents and children when considered as potential participants in studies incorporating new diabetes technologies. As yet, however, relatively little is known about how potential participants feel about participating in randomized trials of diabetes technologies in newly diagnosed youth with type 1 diabetes. The purpose of the present study was to gather qualitative information from parents and children with type 1 diabetes about their experiences of participation in an ongoing randomized study (multiple daily injection [MDI] vs. continuous subcutaneous insulin infusion [CSII]) in which recruitment took place immediately after diagnosis. In addition to informing future study design through a deeper understanding of factors affecting potential participants in randomized trials incorporating new diabetes technology, the present work can be considered as part of wider efforts to explore psychosocial aspects of technology usage in diabetes care and parents' experiences of caring for children using diabetes technologies.^12–14^

## Methods

Focus groups were conducted with parents and children with type 1 diabetes in four pediatric outpatient clinics based in large regional hospitals in England. Participants were recruited on the basis of their involvement with an ongoing study, which involves randomization to either MDI or CSII pump therapy immediately after diagnosis. A heterogeneous composition of parents and children, and of participants and nonparticipants in the ongoing study, was chosen to elicit a variety of perspectives and experiential knowledge (e.g., knowledge arising from experience of diagnosis as a child or a parent).^15^ Each discussion was facilitated by members of the study team (C.F. for focus groups 1–2, M.T. and J.A. for groups 3–4), using a semistructured topic schedule devised by the study team in light of preceding scientific literature.^1–8,10^ Focus group discussions were digitally recorded (with participants' consent), independently transcribed by a trusted agency, and analyzed using a thematic analysis approach.^16^

## Results

The four focus groups (FG1–FG4) averaged 58 min (53–68 min) and included between 8 and 12 focus group participants, with an average of 10 focus group participants (on average 6 parents and 4 children) with a range of demographic characteristics (Table 1). The majority of focus group participants (*n* = 32) had chosen to participate in the ongoing study, but a minority (*n* = 7) had chosen not to participate. Three principal themes—recruitment, randomization, and peer support—were identified through analysis of the focus group transcripts, relating to specific aspects of participants' previous experience of participation in a recent randomized trial. These themes are described below with anonymized quotations from parents and children from the four focus groups (FG1–FG4).

**Table 1.** Focus Group Participants

		*Participation in trial*		*Status*		*Gender*		*Age category*				
*Focus group*	*No. of focus group participants*	*Y*	*N*	*Parent*	*Child*	*Male*	*Female*	*0–9*	*10–18*	*19–35*	*36–52*	*53+*
FG1	9	9	0	5	4	4	5	2	2	1	4	0
FG2	12	12	0	7	5	4	8	0	5	0	7	0
FG3	8	4	4	4	4	3	5	0	4	2	2	0
FG4	10	7	3	6	4	2	8	0	4	1	3	2
Total	39	32	7	22	17	11	24	2	15	5	16	2

### Recruitment

Participants in all focus groups acknowledged the emotional challenges and stress surrounding diagnosis, and consequently the difficulties of signing up to a study and understanding its pros and cons at the same time as coping with significant informational burdens arising from diagnosis. In this context, one parent remarked:

It is a bit of a shock when it's that soon … We had to agree within 10 days of diagnosis and your head's still spinning from the shock of the diagnosis really.

FG1

Other parents, however, suggested that it was good to approach patients early, either because the possibility of being randomized to pump therapy was a possible “light at the end of the tunnel” in the midst of a welter of confusing new information (FG4) or because there was more time to process information in the initial hospital stay than when children had returned home later: “when [we were] in hospital … there's time to sit around and read the stuff and take it all in” (FG3). As such, there are clearly important differences in individual preferences and capacity to absorb complex information.

Some participants suggested that more detailed discussion of the pros and cons of the study would have been useful in the initial stages. In FG2, for example, one parent described the apparent unwillingness of staff to state clearly the purposes of the study and the possible pros and cons:

[W]e were asked as soon as we'd been diagnosed whether we wanted to participate in a study which would have a 50/50 chance of going on a pump or staying on injections, and then when we asked which one's better, a lot of people in the medical profession couldn't tell us one way or the other … [T]hey're very careful about not overloading you with too much information … but if you need a decision … [you're] just going to have to jump in feet first and give a little bit more information.

In light of this, participants suggested that a personal approach could assist understanding: “I think you're so bamboozled, I'm not sure what you could do to unbamboozle [sic] the whole situation … a nice chat might tip the balance either way” (FG2). In this context, many participants highlighted their preference for personal interactions with medical staff as opposed to other resources such as study leaflets or multimedia. For example, one parent stated that

I think when you're in that situation with a poorly child, you just take whatever the doctor says … Wasn't there a DVD or something to watch? … I don't think we've ever watched it.

FG1

Parents mentioned the additional difficulty of attempting to involve children in decision-making while acknowledging their relative lack of understanding of research and its importance: “Most children don't know what … a medical trial or research trial is so … They're just taking in the diabetes and I don't think they would understand what is meant” (FG4). Another parent in the same group suggested that it should not be assumed that children themselves should decide whether to participate or not, seeming to imply that while he thought his child was able to make a good decision and withstand the possible disappointment of randomization, other children may not:

No disrespect, I'm talking about the general public now, if I was the father of another child … dare I say it, I wouldn't let it be the child's decision, in some cases.

By contrast, another parent (also in FG4) stated: “It sounds like we all left the decision up to the child, at the end of the day. You can only tell [them] what you think the benefits, the pros and cons are.” It should be noted however that earlier in this group discussion, it appeared that two children at least would not have participated without their parents' encouragement, and parents in this group also suggested that children do not understand what research is: “I just don't think he would have understood it all.” In FG1, one child described themselves as “scared” about signing up to the study, and that it was their parents' decision rather than theirs. Another child in the same group agreed, stating that her parents had decided that she would participate since there was “an overload of information” making it hard for her “to understand what was happening.” Clearly there is a tension between including children in decision-making, on one hand, and overloading them with stressful information and responsibilities, on the other hand.

### Randomization

Many parents expressed significant concerns about this aspect of their participation in the MDI versus CSII trial, describing the potential disappointment of being randomized to MDI as an additional burden to the informational challenges arising from diabetes. One parent in FG1 described the experience as follows:

[The] flip of the coin came and [our daughter] ended up on injections for 14 months, so she was disappointed she wasn't going to onto a pump, because the way it was sold to us it would make [child's name]'s life so much easier.

One parent described the randomization process as “frustrating”, since you could “land either way”:

It would have been better to have a choice, I understand why not, but have a positive choice … rather than, I'll go in and see where the dice lands … on top of everything else it was just a little bit more pressure.

FG2

In the specific trial they had experienced, participants' frustration and disappointment seem to have risen from a general assumption, facilitated perhaps by discussions with staff regarding the benefits of the CSII arm, that the pump would be a significantly better treatment option than MDI. In FG4, several parents highlighted the need for trial staff to demonstrate equipoise during recruitment. Specifically, these parents suggested that it would be better if the medical staff described the benefits of the pump in a “dull” manner to manage expectations and avoid raising (and possibly then dashing) participants' hopes of obtaining this arm in randomization:

[D]on't make it out to be as good as maybe what it is, so then if you don't get it, you're not as disappointed … [W]hen you get [the pump], then tell me how good it is, but until I get it, [I] don't want to know.

Parents also emphasized the importance of equipoise in terms of issues arising at the end of the study for those randomized to the “technological” arm (i.e., the CSII arm in the previous study), when participants may have to return the new technology having become aware of its benefits. One parent in FG4 expressed this concern with regard to worse glycemic control: “if the research is based on getting such great control for 2 years, woohoo, well, I've had 2 years of great control, and then what?” A parent in FG1 suggested that children's reaction to this change might well be age-dependent: “I mean, children are tolerant, they just take it in their stride, but a teenager would really throw their toys out [of] the pram.” More positively, some parents suggested that the eventual difficulties of ceasing technology usage could be eased with more upfront discussion. As one parent in FG2 stated:

Perhaps the more discussion the better, about the longer term … not just about the immediate challenges of managing the system …, but also what happens at the end of the study.

Despite these concerns, there was a pragmatic recognition from most parents that randomization is necessary for research: “Needless to say the coin went up in the air and we stayed with the injections, which is the way it works” (FG2). The same parent went on to state that, in hindsight, the MDI arm was a useful experience:

And actually with hindsight, I'm really glad that we had a year of injections, because I think that was very beneficial, and I think we learned an awful lot doing that.

As such, parents' initial preference for a particular arm of a given study may be overcome once benefits of the other arm become apparent.

Moreover, some participants initially wished to be randomized to MDI because the pump arm was seen as more technically challenging. Thus, one child stated: “Just, like, it seemed easier going on injections, because I knew more about that than I did the pump” (FG1). Likewise, those who did use a pump acknowledged that it was not necessarily an “easier” option, since it required new skills, in addition to raising issues of visibility. One child whispered in her mother's ear that (as the mother subsequently related) “the thing she finds hardest is people asking her what her pump is, and you know, what's that under your clothes … and she really cannot cope with that at the moment” (FG1). Another child (speaking in FG2) noted that they already had to carry quite a lot of things to school and that additional burdens arising from diabetes care were frustrating:

Well, I do have to bring quite a lot of things to school but … carrying round the injections … and stuff, I have to bring a bag and an extra bag on school trips. An extra bag everywhere and it can be quite annoying.

Overall, there was recognition that no new technology can be a panacea for all ills and will require some additional investment in training and effort. These findings may be significant in light of future trials involving yet more sophisticated closed-loop systems and in which equipoise regarding the two study arms may be yet more imperative (see Discussion).

### Peer support

When discussing support offered to them during trial participation, parents uniformly emphasized the importance of being able to contact clinicians directly for advice and support both by e-mail and telephone. One parent in FG1 stated this view as follows: “it comes down to trust and, you know, when you realise that somebody is [available] on the phone all the time [and] that actually you can do anything with that support. It's knowing that support is there.”

Others, however, suggested that medical staff may try to be “as impartial as possible and not try to influence which road someone goes down” (FG2) and therefore that support and advice from other parents and children would be valuable in addition to clinical advice from healthcare staff. This was often discussed in terms of the ability to access a pool of previous participants who were willing to be contacted by new participants, as one parent in FG3 stated:

I think it's actually nice to see, I think as well from a parent's point of view, an actual person and kind of someone who's been through exactly the same that you've been through.

Another participant in FG3 noted that they would be happy to form part of such a pool of experienced participants themselves, on the basis of their own experience of the lack of such a system:

That [initial] four day period is awful, and I would have loved to have seen someone who had been previously diagnosed and sort of come in as a volunteer and say, we went through this and we done that and just take away some of that doctor talking to me, telling me it's going to be okay, because I believed [the doctor but] it's an awful time.

One parent in FG2 suggested that children be included in the pool of “buddies”, since “as we all know, kids speak to kids in a different language than kids to adults, they relate to each other, so it may help the child if there's any nervousness there.” In this context, a child in FG2 stated: “I think it would probably help if I could talk to someone who had done it before to, like, reassure me”. There are potential ethical issues regarding child-to-child networks, but it seems clear that there is significant appetite for a peer support or “buddy” system, which is a more active and interactive resource than websites, videos, and leaflets. In this context, one parent in FG2 stated explicitly that the existence of such systems would encourage participation in research studies: “If that was something that you were able to build in, I think it would be quite a draw, actually.”

## Discussion

Participants in all four groups highlighted challenges arising from children's potential study participation soon after diagnosis, with particular reference to issues of recruitment and randomization—challenges that led participants to suggest a need for peer support during trials.

In terms of recruitment, participants generally saw diagnosis as a stressful time characterized by information overload, but disagreed regarding both the best time to approach families for recruitment and the child's role in decision-making. However, a consensus emerged to the effect that more personal interactions with healthcare professionals presented the best opportunity to alleviate participants' concerns regarding participation.

In terms of randomization, participants highlighted the potential disappointment arising from allocation to the control arm (in this case, MDI as opposed to CSII), especially in cases in which research staff had failed to display equipoise regarding both trial arms. Participants saw equipoise as important not just in terms of minimizing disappointment for those allocated to the control arm but also for those randomized to the new technology arm (in this case, CSII), since new technologies may be more difficult to use and often have to be surrendered at the end of the trial. In light of these challenges, both parents and children emphasized the need for peer support systems to supplement clinical support, with some participants also suggesting that they would be happy to become peer supporters themselves.

These findings resonate with previous work on challenges surrounding research participation, including both participants' limited understandings of research procedures and the potential for disappointment arising from randomization,^2–7^ and findings on psychosocial challenges arising from diagnosis of type 1 diabetes in youth.^8–11^ The strong focus on personal interaction between trial participants and medical staff echoes previous work that prioritizes personal interaction over multimedia approaches (e.g., videos or computerized presentations), or enhanced consent forms in terms of improving participants' understanding of medical research,^17^ and provides additional support for investment in staffing to provide sufficient personnel for intensive personalized engagements. Furthermore, participants' expressions of their desire for peer support systems are in line with previous research demonstrating how such systems can constitute a powerful adjunct to clinical support when patients are not in contact with medical staff,^18,19^ although research also highlights the need to strike a balance between the independence of peer support networks and the possibility of harmful advice being propagated without clinical moderation.^18^ Ethical issues may also rise in the context of children's expressed desire for child-to-child peer networks alongside, or instead of, parent-to-parent conversations.

In addition, our findings contribute a nuanced exploration of the complexities of technology usage in diabetes studies, thus reinforcing the well-attested need for equipoise in trials.^20^ Participants' concerns regarding randomization ranged beyond the predictable initial disappointment that could arise for those allocated to the control arm, and the equally predictable eventual disappointment for those in the other arm who have to surrender new forms of technology at the end of the study. While clinicians and technologists may be prone to assuming that all or most participants will wish to be allocated to the new technology arm, several participants in our focus groups described the benefits they derived from allocation to the MDI control arm, and in some cases stated that they preferred this arm because of the technical challenges posed by pump usage. Moreover, reflecting previous research on the unpredictability of technology use^21^ and an emerging awareness of the links between diabetes stigma and the aesthetics of medical devices,^22^ one child voiced her discomfort at the social attention generated by her use of a pump that was visible to others, while another discussed their reluctance to carry additional diabetes equipment to school. Thus, equipoise in trials of new diabetes technologies should not only be regarded as a standard aspect of recruitment but should be founded on an in-depth awareness of the complexities of patient interactions with diabetes technology. These complexities are likely to present themselves with yet greater urgency in future with regard to new closed-loop systems (also referred to as “artificial pancreas”, “bionic pancreas”, or “artificial beta cell” systems), which require users to interact with multiple devices—typically, a continuous glucose monitor, smartphone-mounted control algorithm, and wearable insulin pump.^14,23–24^ In trials involving these and other new diabetes technologies, researchers should consider using a range of trial design alternatives, including delayed treatment and crossover designs, to minimize anticipated stress arising from randomization and therefore maximize the likelihood of participation.

## Strengths and Limitations of This Study

We used a qualitative, in-depth focus group approach involving 39 participants, giving a detailed understanding of the experiences of parents and youth with newly diagnosed diabetes. Participants also highlighted the need for intensive personalized engagement with healthcare professionals and equipoise regarding study arms. This research also showed that parents and children placed strong emphasis on the importance of “buddy” systems for peer support. However, only a small minority of focus group participants had chosen not to participate in the ongoing trial. Consequently, this study did not explore in depth the views of parents and children who opted not to participate in the ongoing randomized trial. Ideally, future research would foreground and investigate the views of nonparticipants to identify further barriers to research participation, although ethical issues may arise in this context (e.g., pursuing individuals and families who have already opted out of research to seek consent for further research participation).^25^ Future research could also usefully compare baseline expectations with the details of participant involvement during and after studies of new diabetes technologies, to clarify which specific factors may have contributed to positive and/or negative experiences.

## Conclusions

In conclusion, participants expressed a number of concerns that arise with regard to the research participation of newly diagnosed youth with type 1 diabetes, but also emphasized the way in which these concerns could be alleviated by more intensive personal interaction with healthcare professionals and members of peer support networks. Our findings also highlight the importance of equipoise regarding both arms of studies involving new diabetes technologies. By investing in these aspects of trials, research teams will be able to manage participants' concerns more effectively and thus minimize the anxiety arising from research participation in studies taking place soon after diagnosis.
